# Evaluation of a prototype machine learning tool to semi-automate data extraction for systematic literature reviews

**DOI:** 10.1186/s13643-023-02351-w

**Published:** 2023-10-06

**Authors:** Antonia Panayi, Katherine Ward, Amir Benhadji-Schaff, A Santiago Ibanez-Lopez, Andrew Xia, Regina Barzilay

**Affiliations:** 1 Takeda Pharmaceuticals International AG, Thurgauerstrasse 130, 8152 Glattpark-Opfikon, Zurich, Switzerland; 2 Oxford PharmaGenesis, Oxford, UK; 3 Takeda Pharmaceuticals U.S.A., Inc, Cambridge, MA USA; 4 Massachusetts Institute of Technology, Cambridge, MA USA

**Keywords:** Evidence-based practice, Information science, Information storage and retrieval, Methods, Systematic reviews as topic

## Abstract

**Background:**

Evidence-based medicine requires synthesis of research through rigorous and time-intensive systematic literature reviews (SLRs), with significant resource expenditure for data extraction from scientific publications. Machine learning may enable the timely completion of SLRs and reduce errors by automating data identification and extraction.

**Methods:**

We evaluated the use of machine learning to extract data from publications related to SLRs in oncology (SLR 1) and Fabry disease (SLR 2). SLR 1 predominantly contained interventional studies and SLR 2 observational studies.

Predefined key terms and data were manually annotated to train and test bidirectional encoder representations from transformers (BERT) and bidirectional long-short-term memory machine learning models. Using human annotation as a reference, we assessed the ability of the models to identify biomedical terms of interest (entities) and their relations. We also pretrained BERT on a corpus of 100,000 open access clinical publications and/or enhanced context-dependent entity classification with a conditional random field (CRF) model.

Performance was measured using the F_1_ score, a metric that combines precision and recall. We defined successful matches as partial overlap of entities of the same type.

**Results:**

For entity recognition, the pretrained BERT+CRF model had the best performance, with an F_1_ score of 73% in SLR 1 and 70% in SLR 2. Entity types identified with the highest accuracy were metrics for progression-free survival (SLR 1, F_1_ score 88%) or for patient age (SLR 2, F_1_ score 82%). Treatment arm dosage was identified less successfully (F_1_ scores 60% [SLR 1] and 49% [SLR 2]). The best-performing model for relation extraction, pretrained BERT relation classification, exhibited F_1_ scores higher than 90% in cases with at least 80 relation examples for a pair of related entity types.

**Conclusions:**

The performance of BERT is enhanced by pretraining with biomedical literature and by combining with a CRF model. With refinement, machine learning may assist with manual data extraction for SLRs.

**Supplementary Information:**

The online version contains supplementary material available at 10.1186/s13643-023-02351-w.

## Background

Systematic literature reviews (SLRs) synthesise and critically appraise available evidence, facilitating evidence-based medicine. Evidence-based medicine is the use of the best evidence in making decisions about the care of patients [1], and high-quality SLRs provide the highest level of research evidence; they are commonly used to assess the clinical efficacy of medications or to determine burden of disease [2]. The total number of SLRs conducted has grown substantially in recent years: the International Prospective Register of Systematic Reviews (PROSPERO) registered 284 SLRs in 2011, rising to over 100,000 by the end of 2020 [3].

Owing to the increasing velocity of research output, SLRs must assess more literature than ever before. This, in combination with their inherent rigour, makes these reviews time-intensive, with significant resources spent on data extraction. In a study of 195 records analysed in the PROSPERO registry, the mean length of time taken to conduct an SLR was 67.3 weeks (standard deviation [SD] 31.0 weeks, range 6–186 weeks) [4], with an economic analysis estimating the cost of each SLR at US $141,195 [5]. In addition, SLRs rely on manual data extraction, which makes them prone to errors despite best-practice methods: for example, an analysis of 34 Cochrane SLRs found that 20 contained errors [6].

Automating aspects of the SLR process may be a way to accelerate the conduct of the SLR and to reduce the potential errors in these reviews. Numerous methods have been developed to these ends, but relatively few have focused efforts on the data extraction process [7]. This process includes systematic identification of relevant data from the literature, and, according to the Cochrane Handbook, this identification should be conducted by two independent reviewers to ensure accuracy [8]. Key techniques for automating this process are natural language processing (NLP), which seeks to interpret human language, and machine learning, a technique that can find patterns in data and be applied to NLP.

ExaCT [9] and RobotReviewer [10, 11] ushered the use of machine learning NLP to extract data from clinical trial publications; however, new machine learning methods have been developed since their introduction. Zhang et al. [12] and Golinelli et al. [13] used newer methods to classify whole sentences as relating to categories such as participants, interventions, comparators, or outcomes (PICO). A more precise classification of phrases, which described a wider range of categories, was achieved by Mutinda et al. [14] in clinical trials related to breast cancer; yet, despite good performance, the study extracted entities from abstracts only, did not identify relations between entities, and used a tool that could consider studies with two arms only and could not extract subgroup information. Furthermore, key information, such as drug dosage or study design, was not captured. We propose to solve these limitations with the prototype tool presented in this study.

The state of the art in NLP is centred on the use of language models called transformers, and a popular such model is the bidirectional encoder representations from transformers (BERT) [15]. These models are pretrained on massive amounts of general text, which enables them to have statistical knowledge of how language works. A key characteristic of models such as BERT is their ability to learn the meanings of a word based on its surrounding words (i.e. its context), encoding meanings that can be used as inputs for other models, such as linear or conditional random field (CRF) models [16].

To address the facts that scientific and medical literature uses a specific vocabulary, and that complex relationships often exist between biomedical terms, BERT can be pretrained on biomedical text (as done for the specialised BioBERT model) [17]. Pretrained BERT models can then be fine-tuned to improve performance on specific tasks, including the identification of terms (named-entity recognition) and relationships (relation extraction) specific to, for example, a particular disease of interest.

The objective of this study was to use machine learning to develop a prototype tool that can identify and extract data from scientific and medical literature with the goal of reducing errors and enabling timely completion of SLRs.

## Methods

**Fig. 1:**
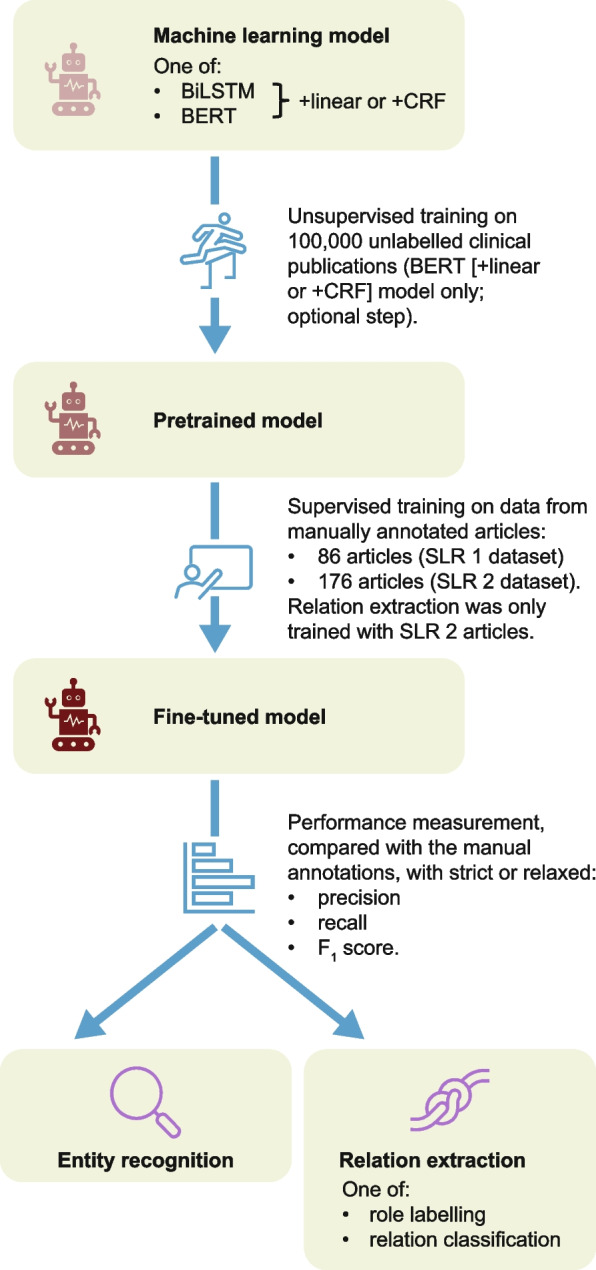
Our development process for refining language models to perform entity recognition and relation extraction. BERT bidirectional encoder representations from transformers, BiLSTM bidirectional long-short-term memory, CRF conditional random field, SLR, systematic literature review

Our method (Fig. 1) performs two classification tasks:

1. named-entity recognition to identify the type of the desired entities
2. relation extraction (through role labelling or relation classification) to identify relations between the entities, using the types extracted by named-entity recognition.

### Model selection and pretraining

Our baseline language model was the original BERT, a transformers-based general language model introduced by Devlin et al. (2018), of which we used the ‘base’ size [15]. For some tests, BERT was pretrained to learn context-rich word representations in the clinical domain. This was achieved using masked language modelling, a self-supervised learning technique, with a corpus of 100,000 open access clinical publications comprising 832,681 passages of text (obtained from PubMed Central). Pre-training was conducted on 4 Nvidia A100 graphical processing units (GPUs) and took 3–4 days.

### Model fine-tuning

To develop models capable of performing named-entity recognition and relation extraction, a manually annotated dataset was required; the machine learning models were fine-tuned on these annotated data using 4 Nvidia A100 GPUs for 6–10 h. The process of selection and annotation of the datasets is summarised in Supplementary Fig. 1 (Additional file 1), which also recapitulates how we fine-tuned and tested the language models.

#### Dataset selection

We created the dataset from a sample of source articles reviewed by two completed SLRs.

To identify a first suitable published SLR, we ran a search in PubMed for SLR articles published between 2018 and 2020. A shortlist of potential high-quality SLRs was obtained by screening the retrieved articles according to several inclusion criteria: evidence of rigorous methodology (e.g. adherence to best-practice guidelines, including risk of bias analysis), a large associated dataset (i.e. review of a sufficient number of references [> 25] for model training), and a clear description of results and data extraction processes. In addition, we chose an SLR that reviewed clinical trial publications in the therapeutic area of oncology because we wanted the publications to contain standardised outcome measures (e.g. progression-free survival [PFS]). Based on this process, the first SLR [18] was selected; this SLR compared the PFS outcomes that were achieved with treatments for relapsed or refractory multiple myeloma.

To extend the scope of the work, we searched for another SLR with similar rigorous methods. We sought an SLR in Fabry disease because it is a disease area of interest for the sponsor of this work (Takeda) and because this disease contains outcome measures that are more variable than in oncology, which would let us test the effect of this variation on the performance of the language models. Finally, we chose to focus on observational studies to determine the performance of the models on this type of study, as opposed to the interventional studies of the first SLR. As a result of this selection process, we chose a second, ongoing SLR (unpublished), which assessed disease burden and treatment outcomes among patients with Fabry disease, including assessment of renal function using the estimated glomerular filtration rate (eGFR).

To improve the fine-tuning of the models, we expanded the original dataset reviewed in the SLRs to include articles reporting similar data. To find these articles, we first ran PubMed searches using the search strings presented in Supplementary Table 1 (Additional file 1). Extensible mark-up language (XML) versions of the articles were required for the annotation tools; therefore, among the articles returned, we selected those for which we had permission to mine the XMLs (XML for Mining licence). Finally, we selected the 70 most recent articles for the SLR 1 dataset expansion and the 150 articles reporting eGFR data for the SLR 2 dataset expansion.

As a result of this process, SLR 1 was defined as the dataset including 16 randomised clinical trials (RCTs) and observational studies on relapsed or refractory myeloma (the dataset from the first SLR), supplemented with a further 70 clinical trial publications reporting PFS data (the dataset expansion). SLR 2 comprised 26 RCTs and observational studies that reported eGFR data in patients with Fabry disease (the dataset from the second SLR), and 150 observational studies that reported eGFR data in patients without Fabry disease (the dataset expansion).

#### Text annotation

Using web-based tools, we manually annotated the publications in SLRs 1 and 2 with terms of interest (entities) and relationships between them (relations). These publications were manually annotated to train our model to extract information related to study design and clinical outcomes. The annotation process was two-stage: (1) entity recognition to identify entities and (2) relation extraction to identify their relations and to classify their relation type. Before annotation, for consistency, we developed a schema for both entities and relations (presented in Tables 1 and 2). Table 1 lists the types of entities that were annotated, along with their descriptions; Table 2 lists the annotated classes of relations, defined by the type of the relation (‘refers to’ and ‘equivalent’) and the types of the two entities in that relation (first and second arguments).

**Table 1:** Definitions of the entity types

**Entity type**	**Description**	Example entity*
SLR 1		
Arm description	Treatment arm description phrase	‘dexamethasone’
Arm dosage	Amount or frequency of a treatment	‘We treated patients with drug X at 1.3 mg/m^2^’
PFS metric	Metric used to describe PFS	‘median progression-free survival’
PFS result	Numeric measurement associated with a PFS metric	‘10 months’
Study type	Type of study design	‘randomized controlled trial’
Title	Title of the publication	‘A study to investigate multiple myeloma’
Authors	Authors of the publication	‘M Smith’
SLR 2		
Age metric	Metric used to measure the age of patient populations	‘mean (SD) age’
Age number	Numeric measurement associated with an age metric	‘60 years’
Arm description	Treatment arm description phrase	‘dexamethasone’
Arm dosage	Amount or frequency of a treatment	‘We treated patients with drug X at 1.3 mg/m^2^’
eGFR metric	Metric used to describe eGFR	‘mean eGFR’
eGFR number	Numeric measurement associated with an eGFR metric	‘20 mL/min/1.73 m^2^’
eGFR subgroup	Population subgroup	‘Among patients >60 years old’
eGFR time point	Time period over which the metric was measured	‘at 4-month follow-up’
Study type	Type of study design	‘observational study’

*eGFR* Estimated glomerular filtration rate, *PFS* Progression-free survival, *SLR* Systematic literature review

^*^These examples serve to illustrate the definition of the entity types and were not taken from the dataset

**Table 2:** Relation schema

Relation type	First argument	Second argument
SLR 1		
Refers to	Arm description	Arm dosage
Refers to	Arm description	PFS metric
Refers to	PFS metric	PFS result
Equivalent	Any	Any
SLR 2		
Refers to	Arm description	Arm dosage
Refers to	eGFR metric	Age number
Refers to	eGFR metric	eGFR number
Refers to	eGFR metric	eGFR subgroup
Refers to	eGFR metric	eGFR time point
Refers to	eGFR number	eGFR subgroup
Refers to	Age metric	Age number
Equivalent	Any	Any

*eGFR* Estimated glomerular filtration rate, *PFS* Progression-free survival, *SLR* Systematic literature review

Owing to the time-consuming nature of text annotation, we used two web-based annotation tools to optimise the process. SLR 1 was annotated with a tool adapted from ChemIE Turk [19] (itself adapted from Amazon Mechanical Turk [20]) that previously proved successful for the annotation of chemical reaction data [21]. SLR 2 was annotated using the brat rapid annotation tool (BRAT; Supplementary Fig. 2, Additional file 1) [22, 23], which improved on the quality of the relation annotations compared with the tool used for SLR 1; details on the web-based annotation tools can be found in the Supplementary Material. In total, the annotation process took 520–580 h. Given the higher-quality relation annotations produced by BRAT, we decided to use the SLR 2 dataset exclusively to train and evaluate the relation extraction model.

#### Annotated data partition

We partitioned the datasets into a training set (see the Supplementary Material for detail on model training), a validation set (for the adjustment of the parameters of the learning algorithm), and a testing set (for performance measurement). Paragraphs were randomly allocated to these datasets following predetermined ratios (SLR 1: 70%, 16%, 14%; SLR 2: 77%, 11.5%, 11.5%, respectively). This resulted in varying ratios of entities and relations, which were dependent on the content of the paragraphs; detailed statistics on this partition are available in Supplementary Table 2 (Additional file 1).

### Named-entity recognition

Annotated data from SLRs 1 and 2 were used to train, validate, and test BERT for the named-entity recognition task.

#### Entity annotation

To recognise the entity types in SLRs 1 and 2, we fine-tuned the pretrained BERT model using the training dataset. Human annotators identified entities and assigned their types as one of those defined in the entity schema (Table 1) using the inside-outside-beginning (also called BIO) annotation format [24]. The annotation process is described in detail in the Supplementary Material.

#### Tokenisation

In the biological domain, it is common for entities to have long names comprising chains of words, numbers, and morphemes (such as prefixes, suffixes, and word roots): to tackle this problem, words can be segmented. BERT-based models split text into smaller units, called tokens, using a tokeniser. First, the text was divided into individual words by splitting on white space between words and punctuation marks. Next, uncommon words (not present in BERT’s tokeniser vocabulary) were decomposed into smaller sub-words (wordpieces) using the WordPiece algorithm because wordpieces have been shown to improve translation performance on rare words [25]. However, decomposition is often not etymologically accurate: for example, ‘dexamethasone’ is split into the wordpieces ‘dex’, ‘ame’, ‘tha’, ‘son’, and ‘e’, whereas an etymological decomposition would be ‘dexa’ (a blend of ‘deca’ and ‘hexa’), ‘meth’ (short for methyl), ‘a’ (combining vowel for euphony), and ‘sone’ (short for cortisone).

#### Encoding

BERT is a transformer model that consists only of an encoder, which means that BERT encodes its input into an abstract representation (a list of 768 numbers). Following tokenisation, the wordpiece tokens were fed into BERT to encode their meanings, with each meaning defined as the content, context, and location of a wordpiece within their sentence. These contextual representations were provided as inputs to the decoder models (described below) to predict entity types. Only the contextual representation of the first wordpiece of each word was input, following Devlin et al. (2019) [15].

#### Decoding

We tested two decoder models: linear and CRF. The linear model computes the probability for each word to be each of the entity types in Table 1 (and the probability for the word to not be such types), whereas the CRF model computes the probability for a sequence of such entity types to occur for the words in each sentence. This enables the CRF model to ensure consistency between predictions, unlike the simpler linear model. Both models label each word with the entity type with the highest probability, but the more complex CRF architecture needs the Viterbi algorithm [26] to determine the best entity-type sequence for each sentence.

The Supplementary Material provides a detailed description of the encoding and decoding architectures for named-entity recognition.

### Relation extraction

Once entities were identified, we sought to extract relations between them. These types of relationships are important for data extraction in SLRs because different entities may need to be considered together to be understood fully. For example, an eGFR number (e.g. ‘20 mL/min/1.73 m^2^’) would need to be extracted with its corresponding eGFR metric (e.g. ‘mean eGFR’) and the treatment arm description (e.g. the ‘dexamethasone’ arm), for context. The relations, defined in the relation schema (Table 2), consisted of a relation type and two related entities. We experimented with two methods for relation extraction: role labelling and relation classification.

Both methods were trained, validated, and tested with the SLR 2 training, validation, and testing datasets, respectively. The role labelling and the relation classification architectures follow closely that for entity recognition, using as input the contextual representation of the first wordpiece of each word, computed by BERT. However, the entity recognition model provides only one entity for the role labelling method and two for relation classification. As a result, role labelling identifies the entities relating to the provided entity and then their relation type (two steps), whereas relation classification immediately characterises the relation between the two provided entities (one step). Detailed descriptions of the architectures of role labelling and relation classification are provided in the Supplementary Material.

In addition, the two methods restricted their search for relations to entities in proximity of each other. Proximity was defined as a distance between entities, called the context-window size, of three or five sentences, because we found that the distance between entity pairs was at most three sentences in 92% of cases and at most five sentences in 97% of cases.

### Performance measurement

To assess the performance of our model, we compared its performance with a common NLP algorithm, the bidirectional long-short-term memory (BiLSTM) model. The BiLSTM model is context-aware, as is BERT through self-attention, but using a more primitive architecture combining two models (one for previous context, the other for subsequent context). We assessed the performance of several BERT-based models and that of BiLSTM-based models using the manually annotated data as a reference.

The identification of an entity or relation by the models is called a predicted positive, which is deemed correct if the identification matches the annotation. For each model, we report precision, recall (sensitivity), and F_1_ score (the average of precision and recall), defined as

$$\begin{array}{c}\mathrm{precision}=\frac{\mathrm{correctly}\;\mathrm{predicted}\;\mathrm{positives}}{\mathrm{predicted}\;\mathrm{positives}}\\\mathrm{recall}=\frac{\mathrm{correctly}\;\mathrm{predicted}\;\mathrm{positives}}{\mathrm{actual}\;\mathrm{positives}}\\F_1\mathrm{score}=H\left(\mathrm{precision},\;\mathrm{recall}\right)=2\times\frac{\mathrm{precision}\times\mathrm{recall}}{\mathrm{precision}+\mathrm{recall}}\end{array}$$

in which *correctly predicted positives* is the count of the correctly identified entities or relations, *predicted positives* is the count of the identified entities or relations, and *actual positives* is the count of the manually annotated entities or relations. *H* denotes the harmonic mean function used to compute average rates.

As is common practice in the reporting of *F*_1_ scores in medical entity recognition tasks, we also report relaxed *F*_1_ scores, which consider partial entity-type matches as correct, whereby there is partial overlap between the predicted entity type and the manually annotated entity type [27, 28]. This is appropriate because long entity types (such as [treatment] arm dosage) are variable in length and may span more than one sentence, making them difficult to identify with precision. In addition, human reviewers may themselves disagree on the exact boundaries of entities: for example, one reviewer may extract ‘We treated patients with drug X at 1.3 mg/m^2^’ as the arm dosage, whereas another reviewer may extract ‘1.3 mg/m^2^’ instead.

## Results

We measured the performance of the named-entity recognition and the relation extraction tasks separately.

### Named-entity recognition

Testing of alternative entity recognition models revealed varying performance for the identification of key data of interest from the scientific publications. Performance measurements showed that BERT-based models consistently outperformed BiLSTM-based models (Table 3). The addition of a CRF model layer improved the performance of BERT-based models but produced less consistent improvement when combined with BiLSTM-based models, compared with the addition of a linear layer. Pretraining baseline BERT to the biomedical domain further improved performance of BERT-based models across all metrics. Pretrained BERT combined with CRF proved to be the best-performing model, improving relaxed *F*_1_ scores over baseline BERT with CRF by 4.1 percentage points in SLR 1 and 8.8 percentage points in SLR 2.

**Table 3:** Entity recognition performance across machine learning models

Model	Relaxed			Strict		
	Precision, %	Recall, %	*F*_1_ score, %	Precision, %	Recall, %	*F*_1_ score, %
SLR 1						
BiLSTM+linear	68	59	63	46	39	42
BiLSTM+CRF	75	53	62	53	38	44
BERT+linear	67	67	67	46	46	46
BERT+CRF	74	65	69	52	46	49
Pretrained BERT+linear	68	72	70	48	50	49
Pretrained BERT+CRF	74	72	**73**	53	52	**52**
SLR 2						
BiLSTM+linear	69	58	63	47	45	46
BiLSTM+CRF	73	56	63	55	42	48
BERT+linear	59	61	59	44	45	43
BERT+CRF	66	58	61	50	45	46
Pretrained BERT+linear	63	67	64	47	50	48
Pretrained BERT+CRF	70	71	**70**	56	56	**55**

Bold indicates the best-performing model. The 95% confidence intervals for the F_1_ scores are included within ± 0.5 percentage points of the estimates given

*BERT* Bidirectional encoder representations from transformers, *BiLSTM* Bidirectional long-short-term memory, *CRF* Conditional random field, *SLR* Systematic literature review

**Fig. 2:**
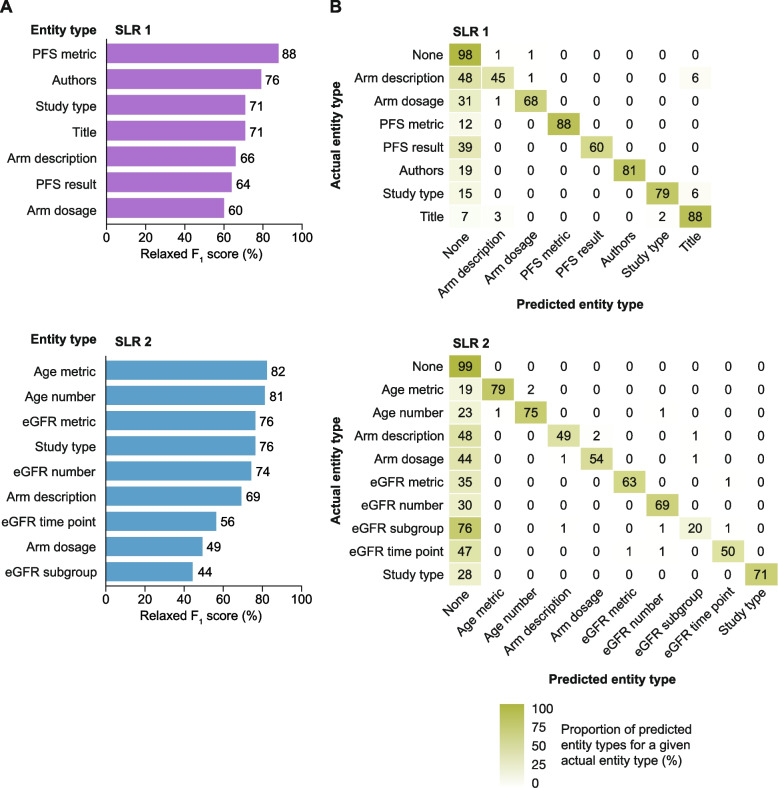
Performance of the pretrained BERT+CRF model across entity types. Panel **A** presents the relaxed *F*_1_ scores and panel **B** compares actual and predicted entity labels using confusion matrices. In **B**, some lines do not sum to 100% owing to rounding. BERT bidirectional encoder representations from transformers, CRF conditional random field, eGFR estimated glomerular filtration rate, PFS progression-free survival SLR systematic literature review

For named-entity recognition broken down by entity type (Fig. 2A), we observed that some entity types were significantly more difficult to predict than others, such as (treatment) arm dosage (relaxed *F*_1_ scores 60% [SLR 1] and 49% [SLR 2]) and eGFR (patient) subgroup (relaxed *F*_1_ score 44% [SLR 2]); this effect was particularly pronounced for strict matching (Supplementary Table 3, Additional file 1), with most of the errors of the model reflecting an inability to label certain entities with their types, rather than mislabelling (Fig. 2B). For example, eGFR subgroup was the worst-performing entity type (as judged by relaxed *F*_1_ score) but was misclassified in only about 3% of labels (mainly as ‘arm description’, ‘eGFR number’, or ‘eGFR time point’). The best-recognised entity types were PFS metric in SLR 1 (relaxed *F*_1_ score 88%) and age metric in SLR 2 (relaxed *F*_1_ score 82%) (Fig. 2A).

### Relation extraction

To extract entity relations, it is necessary to identify the relation type and the two entities in the relations. For example, in a sentence that may contain information on multiple drugs, a (treatment) arm dosage must be linked by a ‘Refers to’ relation to the correct arm description. Relation classification performed better than role labelling at identifying relations for context-window sizes of both three and five sentences, a performance that was further improved with pretraining: pretrained relation classification with a three-sentence context-window size performed best (Table 4).

**Table 4:** Performance of different methods using BERT for relation extraction in the SLR 2 dataset

Model	Three-sentence context window			Five-sentence context window		
	Precision, %	Recall, %	*F*_1_ score, %	Precision, %	Recall, %	*F*_1_ score, %
Role labelling	61	54	57	60	52	56
Relation classification	99	89	93	99	87	92
Pretrained role labelling	64	59	62	62	55	59
Pretrained relation classification	98	91	**95**	98	90	94

The bold F_1_ score indicates the best-performing model. The 95% confidence intervals for the *F*_1_ scores are included within ± 0.5 percentage points of the estimates given.

*BERT* Bidirectional encoded representations from transformers, *SLR* Systematic literature review

Pretrained relation classification was able to identify any entity-type pair (the two entity types of an extracted relation) with more than 80 examples with an F_1_ score of at least 90% (Supplementary Table 4, Additional file 1).

## Discussion

This study assessed machine learning performance in named-entity recognition and relation extraction. For both tasks, we divided words into wordpieces because vocabulary in scientific publications is complex. Then, we computed contextual representations that characterise the content and context of each wordpiece; however, only the contextual representation of the first wordpiece of each word was used for entity recognition and relation extraction. This may have negatively affected the performance of the methods [29]; for example, the classification of the corticosteroid ‘dexamethasone’ using the wordpiece ‘dex’ may have led to spurious classification because this wordpiece may be shared by the central nervous system stimulant ‘dexamfetamine’ and the heart condition ‘dextrocardia’. Using an alternative approach to represent words may improve performance [29]. In addition to this common limitation between the named-entity recognition and relation extraction tasks, we discuss the performance in each individual task next.

### Named-entity recognition

Our data showed that BERT-based models consistently outperformed BiLSTM-based models, probably owing to the ability of BERT to produce high-quality contextual representations for the diverse entities in the biomedical domain. Pretrained BERT combined with CRF proved to be the best-performing model, achieving higher relaxed F_1_ scores than baseline BERT with a CRF layer. This confirms the importance of pretraining baseline general language models to adapt them to a specific domain.

We conducted the pretraining of the models ourselves, which required a large amount of text and processing time. An alternative approach would have been to use pre-existing domain-specific language models, such as BioBERT, ClinicalBERT, SciBERT, and BioMegatron, which are already pretrained. This approach would have saved significant resources [17, 30–32].

Analysis of named-entity recognition performance showed that some entity types are significantly harder to predict than others. This is because some entity types are complex, encompassing short numeric phrases or spanning multiple sentences. In addition, entities of different types may overlap; for example, a treatment arm description may be accompanied by a dosing schedule, making it more difficult for the model to identify them as separate entities. More generally, named-entity recognition in the biomedical domain is challenging owing to the frequent use of abbreviations, homonyms, nested descriptors (entities that belong to different entity types depending on context), spelling differences, and synonyms.

### Relation extraction

Relation extraction performance was better with the relation classification method than with the role labelling method, an observation potentially explained by the more complex task performed by role labelling. Role labelling finds the entities related to a given entity and then classifies the relation, whereas relation classification directly classifies the relation between two given entities. In addition, the poor classification of some entity-type pairs by the relation classification method was due to insufficient numbers of training examples for those pairs. If more than 80 examples were provided, then *F*_1_ scores reached at least 90%. This promising result should encourage further development of the relation extraction method, which is an essential complement to named-entity recognition for providing context to extracted entities.

### Comparison with existing tools

Unlike the previously published tools that extract data from clinical trials [9, 10, 12–14], our method identified relations between entities, for example relating the outcomes of a trial arm to its description. In addition, our named-entity recognition method identifies the precise information needed, unlike previous methods [9, 10, 12, 13], which only classified whole sentences. The most similar study to ours, by Mutinda et al. [14], did identify specific entities but had limitations that we addressed: our tool extracted data from full-text articles, instead of only abstracts [14], and captured key trial information such as study design and drug dosages. In contrast with Mutinda et al., our method could consider studies with three or more arms and subgroup analyses because each outcome was related to the relevant trial arm and subgroup using relation extraction.

The performance of our named-entity recognition was slightly lower than that achieved by Mutinda et al., probably owing to their use of the newer BioBERT [17] and Longformer [33] models; however, our model very rarely misclassified entities (Fig. 2B) whereas this was an issue with the Mutinda et al. method [14]. The authors attributed this issue to the lack of access to full-text publications and to difficulty in differentiating between control and treatment arms, without relation extraction. Although relation extraction solves important limitations for the extraction of data for SLRs, both named-entity extraction and relation extraction face common challenges.

### Common challenges

Human annotation of training data is time-consuming and requires expertise; however, the effort required upfront is later repaid, given that the resultant models can be applied to other tasks requiring extraction of similar data. Maintaining the confidentiality of the data used to train the models was not a concern in this study because we used published data; however, confidential data (such as clinical study reports) should not be used to train publicly available models to maintain data privacy.

Our models were trained using publications, which are generally provided as portable document format (PDF). This heterogenous graphical format makes it difficult to extract text while maintaining the structure of the document. For data extraction, articles should ideally be provided in XML format, but these files are not always readily available and require a licence for use. It is also difficult to extract information from figures and tables, which may contain key data absent from the text, and a limitation of our study is that the model did not extract such information. The GROBID software package [34] converts scientific articles from PDF format to XML format, including figures and tables, and may be helpful for future work.

Several components of SLR development lend themselves to the use of artificial intelligence, such as search-string development, classification of study type, title and abstract screening, extraction of text describing PICO information, risk of bias analysis, data extraction, and data synthesis [7, 9, 11, 35–38]. However, these tasks are not trivial, and current tools (including conversational tools like ChatGPT) are not mature [39]. Owing to the inherent rigour of SLRs, they must continue to be performed with humans ‘in the loop’, a technique that combines the abilities of the machine with human insight and reasoning. Ultimately, it is our goal to develop an end-to-end solution to support reviewers in their quest for improved accuracy and efficiency during the SLR process.

## Conclusions

In this study, we tested the ability of deep-learning language models to extract data of interest from publications, an important step in the SLR development process that would normally be performed by a human analyst. Transformer-based models such as BERT are the current state of the art among language models and are capable of context-rich word representations, effectively capturing the semantics of text.

BERT-based models outperformed others, with further performance gains obtained through domain-specific pretraining, so that our best-performing model demonstrated the ability to recognise key data of interest in scientific texts. With refinement, machine learning may be able to assist with human extraction of data for SLRs, substantially reducing the workload, minimising errors, and decreasing the turnaround time for data synthesis. An immediate goal for automating such data extraction is to develop a tool to perform initial extraction that is then checked by a human; this human–machine tandem may prove to be accurate enough to replace the two independent reviewers recommended by the Cochrane Handbook.

### Supplementary Information

**Additional file 1: **Methodological information on the annotation process and tools and on the computational architectures used for named-entity recognition and relation extraction. **Supplementary Fig. 1.** Our process to create the annotated datasets and use them to train and test the language models. **Supplementary Fig. 2.** An example of a passage of text with entity and relation annotation using BRAT. **Supplementary Table 1.** PubMed searches used to expand the SLR 1 and SLR 2 datasets. **Supplementary Table 2.** Partition between the training, validation, and testing datasets. **Supplementary Table 3.** Entity recognition performance per entity type using pretrained BERT+CRF. **Supplementary Table 4.** Performance of the pretrained relation classification method in the SLR 2 testing dataset across entity-type pairs.

## Data Availability

The data supporting the findings of this study are available within the article and in Additional file 1, except for the entities and relations, which are not available because they were extracted from copyrighted publications. The code used to perform named-entity recognition is available from https://github.com/TakedaGME/MedTrialExtractor/.

## Abbreviations

BERT: Bidirectional encoder representations from transformers
BiLSTM: Bidirectional long-short-term memory
CRF: Conditional random field
eGFR: Estimated glomerular filtration rate
NLP: Natural language processing
PDF: Portable document format
PFS: Progression-free survival
PICO: Participants, interventions, comparators, or outcomes
PROSPERO: The International Prospective Register of Systematic Reviews
RCT: Randomised clinical trial
SD: Standard deviation
SLR: Systematic literature review
XML: Extensible mark-up language
