# Supplementary material for: Evaluation of a prototype machine learning tool to semi-automate data extraction for systematic literature reviews
Source: Syst Rev. 2023 Oct 6;12:187. doi: 10.1186/s13643-023-02351-w (PMC10557215; doi:10.1186/s13643-023-02351-w)
Supplement: Supplementary file 1 — Additional file 1: Methodological information on the annotation process and tools and on the computational architectures used for named-entity recognition and relation extraction. Supplementary Fig. 1. Our process to create the annotated datasets and use them to train and test the language models. Supplementary Fig. 2. An example of a passage of text with entity and relation annotation using BRAT. Supplementary Table 1. PubMed searches used to expand the SLR 1 and SLR 2 datasets. Supplementary Table 2. Partition between the training, validation, and testing datasets. Supplementary Table 3. Entity recognition performance per entity type using pretrained BERT+CRF. Supplementary Table 4. Performance of the pretrained relation classification method in the SLR 2 testing dataset across entity-type pairs. [file 13643_2023_2351_MOESM1_ESM.docx]

# Supplementary material

## Annotation process

Annotators assigned an inside-outside-beginning, or BIO, tag [1] to each word, of the form

\*X*-*type*

in which *X* is B, I, or O: ‘B’ indicates the Beginning of an entity; ‘I’ any subsequent, contiguous word Inside the same entity; and ‘O’ a word Outside of the entities whose types are listed in the entity schema. If *X* is B or I, then *type* is an entity type of the schema; otherwise, if *X* is O, *type* is empty. For example, tagging drug and disease mentions in the sentence, ‘The main objective of the 18-month, randomised, active-controlled ATTRACT study was to assess the effects of migalastat on renal function in patients with Fabry disease.’ results in: ‘The\O main\O objective\O of\O the\O 18\O-\O month\O,\O randomised\O,\O active\O-\O controlled\O ATTRACT\O study\O was\O to\O assess\O the\O effects\O of\O migalastat\B-drug on\O renal\O function\O in\O patients\O with\O Fabry\B-disease disease\I-disease.\O’

## Annotation tools

We used two web-based annotation tools to accelerate the process. The first was adapted from ChemIE Turk [2,3], itself adapted from Amazon Mechanical Turk [4], to enable reviewers with domain expertise to annotate multiple treatment arms in a single paragraph, to reference the location of the paragraph in the original document, and to allow for in-browser access to the portable document format (also called PDF) of the article being annotated. Paragraphs were extracted from the publications and shown to annotators sequentially.

Given that only a few paragraphs from the text contained well-formed examples of named entities from our annotation schema, we employed a set of rules based on publication section and passage-length to show only the paragraphs believed most likely to contain relevant information. We discarded the paragraphs located in the abstract (because they rarely contained sufficient information) and in the discussion section (because they often contained information on unrelated studies or text on treatments based on conjecture). Paragraphs containing fewer than 15 words were also discarded because they often corresponded to figure legends or footers. All the conducted annotation, which was manual, concerned the remaining paragraphs.

Following this manual annotation, the data were manually validated to ensure accuracy and consistency. Annotation took 230–270 hours, with an additional 50 hours for validation with a passage-level accuracy of 75%. Our experience with this annotation tool revealed that it was difficult to go back to make changes and that, if two entities were annotated within the same arm, they were assumed to be either equivalent or related. Therefore, we chose to test the brat rapid annotation tool (BRAT) [5].

Using BRAT, text passages are shown to the annotator as they would appear in the original document. This ability to see paragraphs in context, combined with the facility to correct previous work, significantly reduced annotation time. First, we manually annotated entity types in 70 articles; however, because this was quite labour-intensive, we then used our entity recognition model to automate the annotation of entity types in another 80 papers. We checked these annotations manually and finally annotated relations in all 150 (70 + 80) articles by hand. In a similar amount of time (240–260 hours), the number of documents annotated and validated with this tool was double that achieved by the first tool.

With BRAT, entity relations could be designated by the annotator explicitly instead of being inferred from the order in which they are introduced in the text (Supplementary Fig. 2). In practice, relations were captured by manually linking the entity types that labelled the entities in the relation. This resulted in an improved relation-to-entity ratio for BRAT (0.86) compared with ChemIE Turk (2.07), reflecting a higher quality in relation annotations.

## Named-entity recognition architectures^[[1]](#footnote-1)^

We employed two named-entity recognition architectures: linear and conditional random field (CRF). Both architectures used a bidirectional encoder representations from transformers (BERT) model [6], which is the transformer encoder described by Vaswani *et al.* (2017) [7]^[[2]](#footnote-2),^ In a given sentence of the articles in the datasets for systematic literature reviews (SLRs) 1 and 2, BERT computes a contextual embedding (called ‘contextual representation’ in the main text) for each wordpiece; $\boldsymbol{h}_{i}$ denotes the contextual embedding of the first wordpiece of the word at position $i$.

### The linear architecture

The linear architecture computes the probabilities of each word belonging to each of the entity types given in table 1A, or to none of them. These scores, called emission scores and denoted by $\boldsymbol{z}_{i}^{\mathrm{lin}}$, are computed by a linear layer of the form $\boldsymbol{x}\to\boldsymbol{Wx}+\boldsymbol{b}$, such that

$$\begin{aligned} \boldsymbol{z}_{i}^{\mathrm{lin}}=\boldsymbol{W}_{\mathrm{lin}}\boldsymbol{h}_{i}+\boldsymbol{b}_{\mathrm{lin}},\boldsymbol{\#}\left( 1 \right) \end{aligned}$$

in which $\boldsymbol{W}_{\text{lin}}$ and $\boldsymbol{b}_{\text{lin}}$ are the weight matrix and the bias vector of the linear layer, respectively. The scores are then transformed into probabilities, $\boldsymbol{p}_{i}^{\mathrm{lin}}$, using the $\mathrm{softmax}$ function.

We labelled the $i$th word with the entity type that had the highest probability in $\boldsymbol{p}_{i}^{\mathrm{lin}}$, and the model was trained on annotated data by minimising cross-entropy loss.

### The CRF architecture

The CRF architecture also computes emission scores, $\boldsymbol{z}_{i}^{\mathrm{CRF}}$, but this architecture differs from the linear one by combining a rectified linear unit ($\mathrm{ReLU}$) activation function with a linear layer, so that

$$\begin{aligned} \boldsymbol{z}_{i}^{\mathrm{CRF}}= \mathrm{ReLU}\left( \boldsymbol{W}_{\mathrm{CRF}}\boldsymbol{h}_{i}+\boldsymbol{b}_{\mathrm{CRF}} \right),\boldsymbol{\#}\left( 2 \right) \end{aligned}$$

in which $\boldsymbol{W}_{\text{CRF}}$ and $\boldsymbol{b}_{\text{CRF}}$ are the weight matrix and the bias vector of the linear layer, respectively. From $\boldsymbol{z}_{i}^{\mathrm{CRF}}$, we extract the component emission score $z_{i,l_{i}}^{\mathrm{CRF}}$, which represents the likelihood of the $i$th word being $l_{i}$. In addition, the CRF computes a transition score, $t_{l_{i},l_{i+1}}$, which measures the probability that any two adjacent words, at positions $i$ and $i+1$, have the entity-type labels $l_{i}$ and $l_{i+1}$. In a given sentence, the transition scores are summed for each interval between words (or between a word and a sentence edge), whereas the emission scores are summed for each word. Next, the summed scores are added to form an overall score, $s(\boldsymbol{l)}$, that measures the likelihood of given entity-type labels $\boldsymbol{l=}\left\{ l_{1}, l_{2},\ldots, l_{n} \right\}$. Formally,

$$\begin{aligned} s\left( \boldsymbol{l} \right)= \sum_{i=0}^{n} t_{l_{i},l_{i+1}}+\sum_{i=1}^{n} z_{i,l_{i}}^{\mathrm{CRF}},\#\left( 3 \right) \end{aligned}$$

in which $n$ is the number of words in the sentence.

Subsequently, the overall score, $s(\boldsymbol{l})$, is converted into a probability, $P(\boldsymbol{l})$, with the softmax function, in the following way:

$$\begin{aligned} P\left( \boldsymbol{l} \right)=\frac{e^{s\left( \boldsymbol{l} \right)}}{\sum_{\boldsymbol{l}^{'}\in L} e^{s\left( \boldsymbol{l}^{'} \right)}},\#\left( 4 \right) \end{aligned}$$

in which $L$ is the set of all possible entity-type labels for the words in the sentence. Finally, we used the Viterbi algorithm [9] to compute the entity types, $\hat{\boldsymbol{l}}$, that maximise $P(\boldsymbol{l})$, such that

$$\begin{aligned} \hat{\boldsymbol{l}}=\underset{\boldsymbol{l}\in L}{\mathrm{argmax}} \left( P\left( \boldsymbol{l} \right) \right).\boldsymbol{\#}\left( 5 \right) \end{aligned}$$

These entity types then labelled the words in the sentence, and the model was trained on annotated data by minimising likelihood loss.

## Relation extraction architectures

To identify relations between entities, we tested the role labelling and the relation classification architectures.

### Role labelling

The role labelling architecture [3] first receives a target entity, $target$, identified by the entity recognition model. Then, a BERT model computes a contextual embedding ($\boldsymbol{h}_{i}$) for the first wordpiece of each word at position $i$ in the sentence that contains the target entity. Given that the target entity may contain multiple such wordpieces, represented by embeddings $\boldsymbol{h}_{s}, \boldsymbol{h}_{s+1}, \ldots, \boldsymbol{h}_{e}$, we compute an aggregate representation, $\boldsymbol{h}_{target}$, for the target entity by max-pooling, such that

$$\begin{aligned} \boldsymbol{h}_{target}=\text{max-pool}\left( \boldsymbol{h}_{s}, \boldsymbol{h}_{s+1}, \ldots, \boldsymbol{h}_{e} \right).\#\left( 6 \right) \end{aligned}$$

Next, we concatenate the remaining embeddings ($\boldsymbol{h}_{i}$) in the sentence with the target entity embedding ($\boldsymbol{h}_{target}$) and a representation of the sentence, $\boldsymbol{h}_{sentence}$, output by BERT, to compute a new embedding, $\boldsymbol{h}_{i}^{\boldsymbol{'}}$, for each word, as

$$\begin{aligned} \boldsymbol{h}_{i}^{\mathbf{'}}=\text{concat}\left( \boldsymbol{h}_{i},\boldsymbol{h}_{target}, \boldsymbol{h}_{sentence} \right),\#\left( 7 \right) \end{aligned}$$

in which $\text{concat}$ denotes the concatenation operation. This embedding is used to compute an emission score for each word using Equation (2), but replacing $\boldsymbol{h}_{i}$ with $\boldsymbol{h}_{i}^{\mathbf{'}}$ and the weight matrix and bias vector with new ones optimised for role labelling.

We used this emission score to classify whether each word was related to the target entity and, if so, the entity type of each such word, following the method described for the CRF architecture but replacing $\boldsymbol{z}_{i}^{\mathrm{CRF}}$ with this score.

### Relation classification

The relation classification architecture [10] first receives two target entities ($target_{1}$ and $target_{2}$) and their sentence ($sentence$) from the entity recognition model. As for role labelling, a BERT model computes a contextual embedding, $\boldsymbol{h}_{i}$, for the first wordpiece of each word in $sentence$, as well as a representation of the sentence itself, $\boldsymbol{h}_{sentence}$. Then, we average the embeddings of the tokens included in the target entities with the arithmetic mean function ($\text{mean}$) and apply an activation function ($\text{tanh}$) and a linear layer, such that

$$\begin{aligned} \boldsymbol{h}_{target_{1}}=\boldsymbol{W}_{\text{rel}}\text{ ∙ tanh}\left( \text{mean}\left( \boldsymbol{h}_{s_{1}}, \boldsymbol{h}_{s_{1}+1}, \ldots, \boldsymbol{h}_{e_{1}} \right) \right)+\boldsymbol{b}_{\text{rel}},\#\left( 8 \right) \end{aligned}$$

$$\begin{aligned} \boldsymbol{h}_{target_{2}}=\boldsymbol{W}_{\text{rel}}\text{ ∙ tanh}\left( \text{mean}\left( \boldsymbol{h}_{s_{2}}, \boldsymbol{h}_{s_{2}+1}, \ldots, \boldsymbol{h}_{e_{2}} \right) \right)+\boldsymbol{b}_{\text{rel}},\#\left( 9 \right) \end{aligned}$$

$$\begin{aligned} \boldsymbol{h}_{sentence}^{\mathbf{'}}=\boldsymbol{W}_{\text{rel’}}\text{ ∙ tanh}\left( \boldsymbol{h}_{sentence} \right)+\boldsymbol{b}_{\text{rel’}},\boldsymbol{\#}\left( 10 \right) \end{aligned}$$

in which the embeddings of the tokens included in $target_{1}$ and $target_{2}$ are denoted by $\boldsymbol{h}_{s_{1}}, \boldsymbol{h}_{s_{1}+1}, \ldots, \boldsymbol{h}_{e_{1}}$ and $\boldsymbol{h}_{s_{2}}, \boldsymbol{h}_{s_{2}+1}, \ldots, \boldsymbol{h}_{e_{2}}$, respectively. In addition, $\boldsymbol{W}_{\text{rel}}$ and $\boldsymbol{W}_{\text{rel’}}$ are the weight matrices, whereas $\boldsymbol{b}_{\text{rel}}$ and $\boldsymbol{b}_{\text{rel’}}$ are the bias vectors of the linear layers that compute the target entities representations, $\boldsymbol{h}_{target_{1}}$ and $\boldsymbol{h}_{target_{2}}$, and the updated sentence representation, $\boldsymbol{h}_{sentence}^{\boldsymbol{'}}$, respectively. The three representations are then concatenated to produce a contextual embedding of the relation, $\boldsymbol{h}_{relation}$, defined by

$$\begin{aligned} \boldsymbol{h}_{relation}=\text{concat}\left( \boldsymbol{h}_{target_{1}},\boldsymbol{h}_{target_{2}}, \boldsymbol{h}_{sentence}^{\mathbf{'}} \right).\boldsymbol{\#}\left( 11 \right) \end{aligned}$$

Next, we calculate the vector $\boldsymbol{p}$ that contains the probabilities that the relation belongs to the relation types defined in table 1B, or to none of them. These probabilities are computed by first applying a linear layer and then the $\text{softmax}$ function to the relation representation ($\boldsymbol{h}_{relation}$), such that

$$\begin{aligned} \boldsymbol{p}_{\mathrm{rel}}=\text{softmax}\left( \boldsymbol{W}_{\text{rel''}}\boldsymbol{h}_{relation}+\boldsymbol{b}_{\text{rel''}} \right),\boldsymbol{\#}\left( 12 \right) \end{aligned}$$

in which $\boldsymbol{W}_{\text{rel''}}$ and $\boldsymbol{b}_{\text{rel''}}$ are the weight matrix and the bias vector, respectively, of the linear layer.

We labelled the relation between the two target entities as the relation type with the highest probability in $\boldsymbol{p}_{\mathrm{rel}}$ (the types of the entities within the relation are provided by the entity recognition model). The relation classification model was trained by minimising cross-entropy loss, with the use of the dropout regularisation method.

## Supplementary figures

**
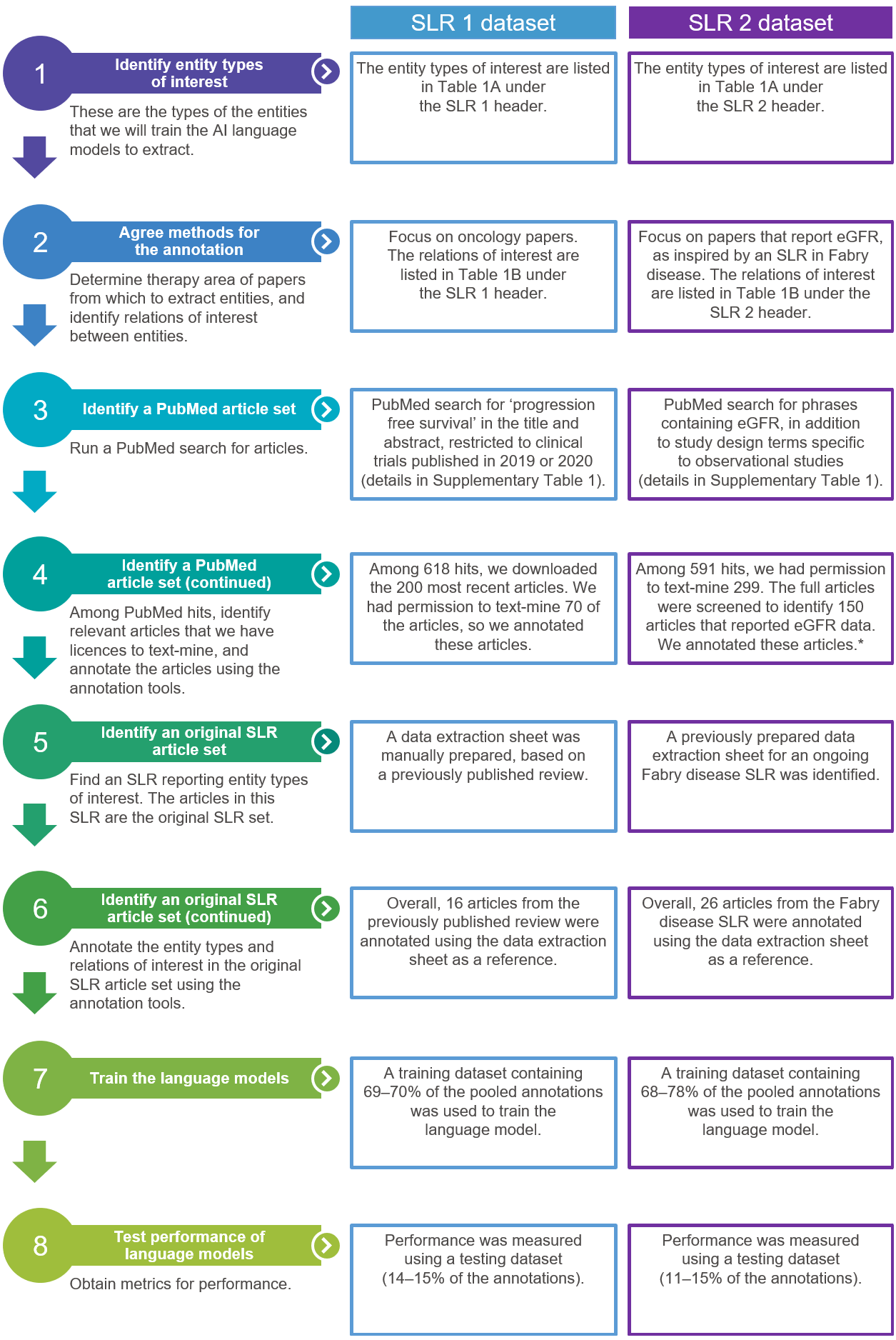
**

**Supplementary Fig. 1** Our process to create the annotated datasets and use them to train and test the language models.

*Entity types were annotated manually in 70 articles; in the remaining 80 articles, we used our entity recognition model to automate the annotation of entity types, which we edited and corrected by hand. Relations were manually annotated in all 150 articles.

AI, artificial intelligence; eGFR, estimated glomerular filtration rate; SLR, systematic literature review.


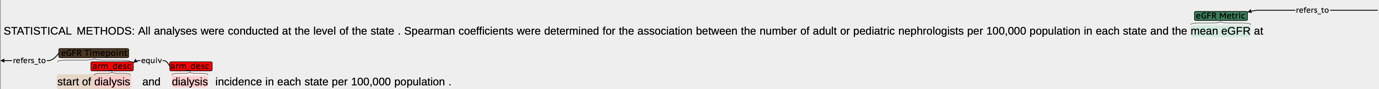
 **Supplementary Fig. 2** An example of a passage of text with entity and relation annotation using BRAT.

BRAT, brat rapid annotation tool.

## Supplementary tables

**Supplementary Table 1** PubMed searches used to expand the SLR 1 and SLR 2 datasets.

SLR, systematic literature review.

**Supplementary Table 2** Partition between the training, validation, and testing datasets.


Percentages in some columns do not sum to 100 owing to rounding.

SLR, systematic literature review.

**Supplementary Table 3** Entity recognition performance per entity type using pretrained BERT+CRF.

Bold blue F_1_ scores indicate the best-recognised entity types.

‘Actual count’ represents the number of manually annotated entities of each given type in the dataset.

BERT, bidirectional encoder representations from transformers; CRF, conditional random field; eGFR, estimated glomerular filtration rate; PFS, progression-free survival; SLR, systematic literature review.

**Supplementary Table 4** Performance of the pretrained relation classification method in the SLR 2 testing dataset across entity-type pairs.

‘Actual count’ represents the number of manually annotated entity pairs of given types in the dataset.

Bold blue F_1_ scores indicate entity-type pairs with actual counts of at least 80.

eGFR, estimated glomerular filtration rate; SLR, systematic literature review.

## References

1. Ramshaw LA, Marcus MP. Text chunking using transformation-based learning. Third Workshop on Very Large Corpora; May 1995, Massachusetts Institute of Technology, Cambridge, MA, USA. Stroudsburg, PA: Association for Computational Linguistics; 1995:82–94.

2. Ibanez-Lopez AS. ChemIE-Turk: Mechanical turk on your own machine for chemical literature annotation [Internet]. GitHub. 2021. <https://github.com/asibanez/chemie-turk>. Accessed 12 May 2021.

3. Guo J, Ibanez-Lopez AS, Gao H, et al. Automated chemical reaction extraction from scientific literature. J Chem Inf Model. 2022;62:2035–45. doi:10.1021/acs.jcim.1c00284

4. Amazon. Amazon Mechanical Turk [Internet]. 2005. <https://www.mturk.com/>. Accessed 2 Nov 2022.

5. Stenetorp P, Pyysalo S, Topić G, et al. brat: a web-based tool for NLP-assisted text annotation. Proceedings of the Demonstrations at the 13th Conference of the European Chapter of the Association for Computational Linguistics; April 2012, Avignon, France. Stroudsburg, PA: Association for Computational Linguistics; 2012:102–7.

6. Devlin J, Chang M, Lee K, et al. BERT: pre-training of deep bidirectional transformers for language understanding. arXiv preprint 2019;arXiv:1810.04805v2. <https://arxiv.org/abs/1810.04805>

7. Vaswani A, Shazeer N, Parmar N, et al. Attention is all you need. Proceedings of the 31st International Conference on Neural Information Processing Systems (NIPS'17); 4–9 December 2017, Long Beach, CA, USA. Red Hook, NY: Curran Associates Inc.; 2017:6000–10.

8. Vaswani A, Bengio S, Brevdo E, et al. Tensor2Tensor for neural machine translation. arXiv preprint 2018;arXiv:1803.07416. <https://arxiv.org/abs/1803.07416>

9. Forney GD. The Viterbi algorithm. Proc IEEE. 1973;61:268–78. doi:10.1109/PROC.1973.9030

10. Wu S, He Y. Enriching pre-trained language model with entity information for relation classification. Proceedings of the 28th ACM International Conference on Information and Knowledge Management; 3–7 November 2019, Beijing, China. New York, NY: Association for Computing Machinery; 2019:2361–4.

1. In the following sections, we follow the convention that sets are typed in capitals, scalars in lowercase, vectors in lowercase bold, and matrices in bold capitals. Variables (which can be sets, scalars, vectors, or matrices) are typed in italics, and the rest is typed in roman. [↑](#footnote-ref-1)
2. The software code for this model is available as the Tensor2Tensor library of Google’s TensorFlow project [8]. [↑](#footnote-ref-2)
